# Factors Associated With Male Breast Cancer Incidence Among Prostate Cancer Survivors: Real World Evidence From Veterans Affairs National Prostate Cancer Data Core

**DOI:** 10.1002/pros.70074

**Published:** 2025-10-08

**Authors:** Erum Z. Whyne, Sung‐Hee Choi, Nisha Unni, Shifa Kanjwal, Jonathan E. Dowell, Haekyung Jeon‐Slaughter

**Affiliations:** ^1^ VA North Texas Health Care System Dallas Texas USA; ^2^ Department of Internal Medicine University of Texas Southwestern Medical Center Dallas Texas USA

**Keywords:** cardiovascular disease, furosemide, male breast cancer, prostate cancer, spironolactone, veterans

## Abstract

**Background:**

While male breast cancer incidence is rare, veteran status is found to be associated with increased risk, for incidence, a higher prevalence of male breast cancer patients was observed among male veteran prostate cancer survivors. This study leveraged the existing large‐scale Veterans Affairs (VA) Prostate Cancer Data Core and examined factors associated with increased risk of male breast cancer incidence in veterans with prior prostate cancer diagnoses.

**Methods:**

A retrospective cohort study of 1.3 million male veterans treated for prostate cancer at VA hospitals was conducted using the VA Prostate Cancer Data Core. Of these, 11,327 (0.86%) were newly diagnosed with male breast cancer on average 5.4 years post prostate cancer diagnosis.

**Results:**

Multivariate Cox and competing risk model results found that younger onset age of prostate cancer (hazard ratio [HR] 0.97, 95% confidence interval [CI] 0.97–0.98), metastasized prostate cancer (HR 2.03, 95% CI 1.90–2.17), being Non‐Hispanic (N‐H) Black (HR 1.10, 95% CI: 1.05–1.15), radiation (HR 1.06, 95% CI: 1.02–1.11) and androgen deprivation therapy (ADT; HR 1.24, 95% CI 1.17–1.32) were associated with significantly increased risk of male breast cancer diagnosis. Prolonged use of cardiovascular disease (CVD) medications, furosemide (HR 1.51, 95% CI 1.39–1.63), spironolactone (HR 1.36; 95% CI 1.15–1.61), and digoxin (HR 1.50, 95% CI: 1.29–1.72), significantly increased risk for male breast cancer incidence.

**Conclusions:**

Younger age onset of prostate cancer, metastasized prostate cancer, prolonged use of CVD medications, radiation, and ADT cancer treatment were factors significantly associated with increased risk of being diagnosed with male breast cancer among male veteran prostate cancer survivors. The study findings may shed insights in cardio‐oncology specific risk factors for male breast cancer among prostate cancer survivors.

## 1 Introduction

Male breast cancer is rare, accounting for approximately 1% of all breast cancer diagnoses [1, 2]. In the United States (US), about 2,800 new cases of male breast cancer and 510 deaths are estimated to occur in 2025 [3]. Given the scarcity of male breast cancer patients, the vast majority of research and guidelines in breast cancer has focused on and extrapolated from female populations. While mortality rates for females with breast cancer have declined, male breast cancer survival rates have not changed in over 30 years [4], resulting in male breast cancer patients having a 19% higher mortality rate than women [5]. When focusing on veterans diagnosed with breast cancer, mortality rates of male veterans are twice as high as female veterans, 18% versus 9% [6], emphasizing the need for further research in male breast cancer and particularly in veterans.

Male breast cancer distinctly differs from female breast cancer in disease characteristics [2]. One‐third of all male breast cancers are secondary cancers, with prostate cancer (PCa) being the most common primary cancer [7]. Patients diagnosed with PCa have a significant increased risk of subsequently being diagnosed with male breast cancer [8]. One known mechanism for development of male breast cancer is breast enlargement, known as gynecomastia, at older ages. Changes in sex hormones—increased estrogen and decreased testosterones—among males may contribute to gynecomastia. In PCa patients, lower testosterone levels are the intended treatment effects of hormone therapy (i.e., androgen depravation therapy; ADT), such as Gonadotropin receptor hormone (GnRH) agonists and antagonists, to slow down growth of prostate cancer cells.

GnRH agonists, such as leuprolide, goserelin, and triptorelin, and GnRH antagonists, such as degarelix, are used to treat non‐metastatic PCa patients to lower testosterone by decreasing luteinizing hormone and follicle‐stimulating hormone levels. Other PCa treatment are adrenal androgen receptor inhibitors (i.e., abiraterone) and direct androgen receptor inhibitors (i.e., apalutamide, enzalutamide, darolutamide, bicalutamide [9, 10], and flutamide [11, 12]), which work downstream to lower testosterone activity. Prostate cancer patients treated with ADT, anti androgen medications, and brachytherapy [13, 14, 15] later may be at an elevated risk of gynecomastia.

Furthermore, patients treated with ADT, such as GnRH agonists and antagonists, and anti‐androgen medications, may be at elevated risk of cardiovascular disease (CVD) events, such as heart failure. Cardiovascular medications, such as spironolactone [16, 17], digoxin [18], and furosemide [19], have been linked with increased risk of breast cancer and are common concomitant medications during cancer treatment.

Given male breast cancer is rare, most previous studies have been case reports or small pilot studies. Thus, there is a substantial gap in knowledge and research about risk factors for male breast cancer, particularly due to the scarcity of data. To overcome this limitation, we utilized real world evidence, existing large‐scale Veterans Affairs (VA) Prostate Cancer Data Core built from electronic health records (EHR) data from the Veterans' Health Administration (VHA), the largest integrated health care system in the US.

## 2 Materials and Methods

### 2.1 Data Source

This study utilized data from the VA Prostate Data Core, a data repository of all male veterans diagnosed with prostate cancer who received care at the VHA, extracted from VA Corporate Data Warehouse (CDW) [20]. The VA North Texas Health Care System Institutional Review Board (IRB) approved the study and informed consent was waived for this study.

**Figure 1:**
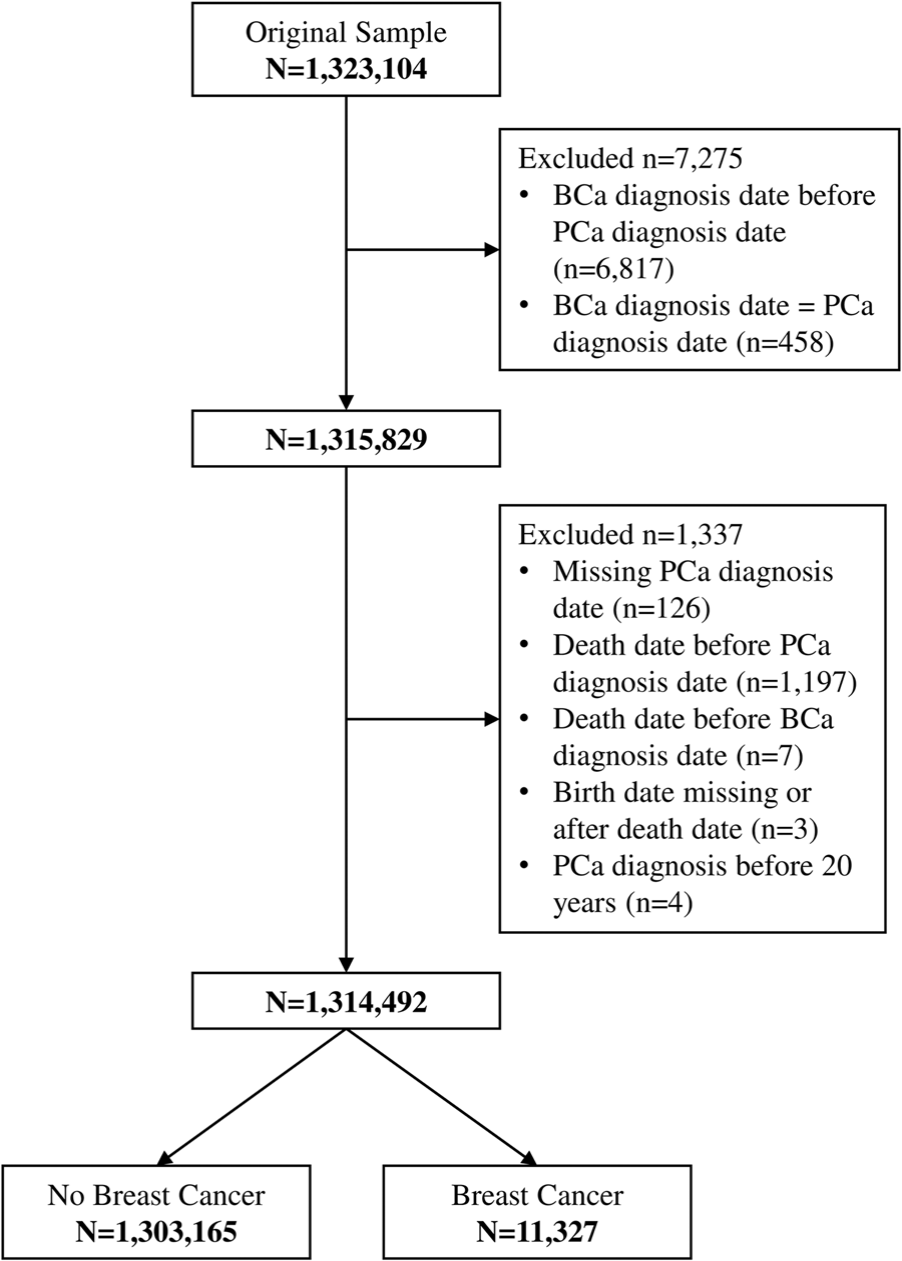
Flow diagram of male prostate cancer patients for final analysis. BCa, breast cancer; PCa, prostate cancer.

The initial VA Prostate Data Core consisted of 1,323,104 male veterans diagnosed with prostate cancer and treated at VHA between January 1, 2000 and March 12, 2024. Of these, the study excluded male veterans diagnosed with breast cancer before or at the same time as PCa diagnosis (*n* = 7275) and with data discrepancies in diagnosis dates (*n* = 1337), yielding a final sample size of 1,314,492 (See Figure 1). The majority were Non‐Hispanic (N‐H) White (68.84%) on average 71.74 years old (SD = 9.26) at time of PCa diagnosis, and with a median survival of 6 years. Approximately 7% (*n* = 95,343) had metastatic PCa diagnosis, 3% (*n* = 39,467) had castrate resistant prostate cancer (Table 1), and < 1% (0.86%, *n* = 11,327) were diagnosed with male breast cancer after their initial PCa diagnosis.

**Table 1:** Descriptive statistics–male veteran patients with prostate cancer (*N* = 1,314,492).

Characteristics		Total sample	No breast cancer	Breast cancer	*p* value^1^
		*N* = 1,314,492	*n* = 1,303,165	*n* = 11,327	
Age at PCa Dx	Mean ± SD	71.74 ± 9.26	71.78 ± 9.25	67.30 ± 8.91	< 0.0001
Age at BCa Dx	Mean ± SD	72.72 ± 9.19	–	72.72 ± 9.19	–
Age at death	Mean ± SD	81.29 ± 9.00	81.30 ± 9.00	80.27 ± 9.33	< 0.0001
Race	< 0.0001				
N‐H White	*n* (%)	904,861 (68.84)	896,858 (68.82)	8,003 (70.65)	
N‐H Black	*n* (%)	221,307 (16.84)	218,753 (16.79)	2,554 (22.55)	
Native American	*n* (%)	5,390 (0.41)	5,345 (0.41)	45 (0.40)	
Asian	*n* (%)	6,128 (0.47)	6,096 (0.47)	32 (0.28)	
Hawaiian	*n* (%)	6,960 (0.53)	6,904 (0.53)	56 (0.49)	
Multi race/Unknown	*n* (%)	120,133 (9.14)	119,921 (9.20)	212 (1.87)	
Hispanic	*n* (%)	49,713 (3.78)	49,288 (3.78)	425 (3.75)	
BMI (kg/m^2^)^2^	Mean ± SD	28.39 ± 5.39	28.39 ± 5.39	28.83 ± 5.79	< 0.0001
PSA	Mean ± SD	17.71 ± 226.35	17.77 ± 227.00	12.40 ± 156.10	0.0140
Death	*n* (%)	809,510 (61.58)	802,278 (61.56)	7,232 (63.85)	< 0.0001
Castrate resistant PCa	*n* (%)	39,467 (3.00)	38,664 (2.97)	803 (7.09)	< 0.0001
Metastatic PCa	*n* (%)	95,343 (7.25)	93,801 (7.20)	1,542 (13.61)	< 0.0001
mCRPC	*n* (%)	33,853 (2.58)	33,164 (2.54)	689 (6.08)	< 0.0001
**PCa treatment** 3					
ADT^4^	*n* (%)	225,417 (17.15)	221,597 (17.00)	2,891 (25.52)	< 0.0001
Anti‐androgen treatment^5^	*n* (%)	197,796 (15.05)	194,607 (14.93)	2,403 (21.21)	< 0.0001
Abiraterone	*n* (%)	27,517 (2.09)	27,312 (2.10)	205 (1.81)	0.0343
Platinum chemotherapy	*n* (%)	26,087 (1.98)	25,808 (1.98)	279 (2.46)	0.0002
Radiation therapy^6^	*n* (%)	339,872 (25.86)	335,954 (25.78)	3,918 (34.59)	< 0.0001
**CVD medications** 3					
Furosemide	*n* (%)				< 0.0001
Never		984,250 (74.88)	975,620 (74.87)	8,630 (76.19)	
Initiation after PCa Dx		219,538 (16.70)	217,859 (16.72)	1,679 (14.82)	
Discontinuation after PCa Dx		36,159 (2.75)	35,967 (2.76)	192 (1.70)	
Continuation after PCa Dx		74,545 (5.67)	73,719 (5.66)	826 (7.29)	
Spironolactone	*n* (%)				< 0.0001
Never		1,226,419 (93.30)	1,215,920 (93.31)	10,499 (92.69)	
Initiation after PCa Dx		11,778 (0.90)	11,713 (0.90)	65 (0.57)	
Discontinuation after PCa Dx		64,190 (4.88)	63,575 (4.88)	615 (5.43)	
Continuation after PCa Dx		12,105 (0.92)	11,957 (0.92)	148 (1.31)	
Digoxin	*n* (%)				0.0010
Never		1,240,895 (94.40)	1,230,206 (94.40)	10,689 (94.37)	
Initiation after PCa Dx		10,863 (0.83)	10,789 (0.83)	74 (0.65)	
Discontinuation after PCa Dx		43,555 (3.31)	43,199 (3.31)	356 (3.14)	
Continuation after PCa		19,179 (1.46)	18,971 (1.46)	208 (1.84)	
**Time/duration (years)**					
PCa Dx to BCa Dx	Mean ± SD	5.42 ± 4.75	‐‐‐	5.42 ± 4.75	‐‐‐
	Median (IQR)	4.08 (6.38)	‐‐‐	4.08 (6.38)	‐‐‐
PCa Dx to death	Mean ± SD	7.48 ± 5.78	7.4 ± 5.77	11.3 ± 5.9	< 0.0001
	Median (IQR)	6.33 (8.70)	6.3 (8.7)	11.0 (8.7)	
BCa Dx to death	Mean ± SD	6.12 ± 4.94	‐‐‐	6.12 ± 4.94	‐‐‐
	Median (IQR)	5.06 (7.27)	‐‐‐	5.06 (7.27)	‐‐‐

Abbreviations: ADT, androgen deprivation therapy; BCa, breast cancer; BMI, body mass index; CVD, cardiovascular disease; Dx, diagnosis; IQR, interquartile range; N‐H, non‐Hispanic; PCa, prostate cancer; PSA, prostate‐specific antigen; SD, standard deviation.

^1^ Chi‐square statistics and *t*‐statistics were used to examine group differences in baseline categorical and continuous covariates, respectively, between the no breast cancer and breast cancer groups.

^2^ BMI missing for total sample *n* = 71,718, with *n* = 71,656 in no breast cancer group and *n* = 62 in breast cancer group.

^3^ For breast cancer group, only patients who started treatments/medications before breast cancer diagnosis date are included.

^4^ ADT includes: orchiectomy, leuprolide, goserelin, triptorelin, histrelin, buserelin, degarelix, and relugolix.

^5^ Anti‐androgen treatment includes: cyproterone, flutamide, bicalutamide, nilutamide, enzalutamide, apalutamide, and darolutamide.

^6^ Supporting Information Table 2 includes codes used to identify brachytherapy.

### 2.2 Variables of Interest

Data on demographics (race/ethnicity and age at PCa diagnosis), clinical presentations ‐Body Mass Index (BMI) and Prostate‐Specific Antigen (PSA), prostate cancer treatments, CVD medications, vital status (e.g., date of death), age at breast cancer diagnosis, and duration from prostate cancer to breast cancer diagnosis are presented in Table 1.

PCa diagnosis for patients in the VA Prostate Data Core were identified using International Classification of Diseases (ICD) 9 and 10 diagnosis codes, ICD 9 and 10 procedure codes (prostate biopsies), and patients from the VA Central Cancer Registry (VACCR) with prostate as their primary site of tumor [21]. Breast cancer (Supporting Information Table 1), and certain prostate cancer treatments (radiation‐brachytherapy and ADT‐orchiectomy; Supporting Information Table 2) were defined by the ICD 9 and 10 diagnosis codes, ICD 9 and 10 procedure codes, and Current Procedural Terminology (CPT) codes. Veterans with castrate resistant prostate cancer and/or metastatic PCa were identified using both structured and Natural Language Process (NLP) data.

PCa treatments (ADT, anti‐androgen medications, and radiation‐brachytherapy), spironolactone, furosemide, and digoxin were identified using inpatient, outpatient, and fee‐based VA pharmacy and CPT data. ADT includes orchiectomy, leuprolide, goserelin, triptorelin, histrelin, buserelin, degarelix, and relugolix. Anti‐androgen medications include cyproterone, flutamide, bicalutamide, nilutamide, enzalutamide, apalutamide, and darolutamide. Platinum‐chemotherapy was selected for chemotherapy while other chemotherapy, such as docetaxel and paclitaxel, were considered but not included in the model. Variables were selected in the model following criteria of Akaike Information Criterion (AIC)—a smaller AIC is a better fit—and statistical significance. For duration of spironolactone, furosemide, and digoxin, these were categorized into four groups: (1) Never on medication, (2) initiation after prostate cancer diagnosis (no before PCa/yes after PCa), (3) discontinuation after PCa diagnosis (yes before PCa/no after PCa), and (4) continuation on medication(yes before PCa/yes after PCa; Table 1, Supporting Information Table 3).

### 2.3 Statistical Analysis

Means and standard deviations and frequency and percentages, were reported as descriptive statistics for continuous and categorical variables, respectively. *T*‐statistics and chi‐square statistics were used to test group differences in continuous and categorical variables, respectively, between male prostate cancer patients with and without breast cancer diagnosis.

Cox proportional hazard model was conducted to identify significant risk factors associated with increased risk of male breast cancer. Death is a competing event for breast cancer incidence, meaning breast cancer diagnosis (event of interest) can not be observed if death occurrs before breast cancer diagnosis. Without considering death as a competing event for breast cancer incidence, the hazard of breast cancer incidence is underestimated. Therefore, a Fine‐Gray competing risk model was also performed to conduct a time to breast cancer event analysis including death as a competing event. Time to event was calculated from date of prostate cancer diagnosis until breast cancer diagnosis date, death date, or end of the study (March 12, 2024).

Due to missingness of BMI, *n* = 71,718 (5.46%), it was not included as a covariate in the final model; all other variables had complete data. However, to evaluate the robustness of results, both Cox and Fine‐Gray models were performed with BMI as a covariate as well (See Supporting Information Table 4). Hazard ratios (HRs), sub‐distribution hazard ratios (SHRs), and 95% confidence intervals (CIs) were reported as time to event analysis results. A *p‐value* < 0.05 was considered statistically significant. All analyses were performed using SAS version 9.4 (SAS Institute, Cary, NC) and results were visualized using the “forestplot” package in R software within the VA Informatics and Computing Infrastructure (VINCI).

## 3 Results

Of 1,314,492 male veterans with prostate cancer, < 1% (0.87%, *n* = 11,327) were diagnosed with breast cancer on average within 6 years after PCa diagnosis (Median = 4.08, Mean = 5.42, SD = 4.75). Males diagnosed with breast cancer compared to those without, were significantly younger at the time of their PCa diagnosis (67 years vs 72 years), and survived longer after their PCa diagnosis (11.0 years vs 6.3 years). Additionally, males diagnosed with breast cancer were more likely to have castrate resistant prostate cancer (7.09% vs 2.97%) and metastatic prostate cancer (13.61% vs 7.20%) than the no breast cancer group (Table 1).

Table 2 shows Cox and competing risk model results. The cox model showed that a younger age onset of prostate cancer (HR 0.97, 95% CI 0.97–0.98) and metastasized prostate cancer (HR 2.03, 95% CI 1.90–2.17) increased risk of male breast cancer diagnosis. Compared to N‐H Whites, N‐H Blacks were more likely to develop breast cancer (HR 1.10, 95% CI 1.05–1.15), while Hispanic male veterans (HR 0.85, 95% CI 0.77–0.94), Asians (HR 0.61, 95% CI 0.43–0.86), and multi/unknown race (HR 0.31, 95% CI 0.27–0.36) were less likely. Brachytherapy (HR 1.06, 95% CI 1.02–1.11) and ADT (HR 1.24, 95% CI 1.17–1.32) significantly increased risk of breast cancer diagnosis, while abiraterone significantly decreased risk (HR 0.36, 95% CI 0.31–0.42), and anti‐androgen medications and platinum chemotherapy were not significantly associated (Table 2).

**Table 2:** Multivariable Cox hazard regression and Fine‐Gray sub‐distribution hazards (competing risk) model for male breast cancer in prostate cancer patients.

Variables	Cox			Competing risk		
	HR	95% CI	*p* value	SHR	95% CI	*p* value
Covariates						
Age PCa Dx	0.974	0.972, 0.977	< 0.0001	0.957	0.955, 0.959	< 0.0001
Metastatic PCa Dx	2.029	1.900, 2.166	< 0.0001	1.684	1.565, 1.812	< 0.0001
Race–versus N‐H White						
Native American	0.872	0.652, 1.167	0.3581	0.851	0.635, 1.140	0.2798
Asian	0.608	0.431, 0.858	0.0047	0.628	0.444, 0.889	0.0086
Hawaiian/PI	0.819	0.630, 1.064	0.1344	0.846	0.651, 1.101	0.2135
Hispanic	0.846	0.767, 0.933	0.0008	0.866	0.786, 0.956	0.0042
Multi race/Unknown	0.312	0.272, 0.358	< 0.0001	0.218	0.190, 0.250	< 0.0001
N‐H Black	1.095	1.046, 1.147	0.0001	1.047	0.999, 1.097	0.0547
PCa treatments						
Radiation therapy	1.059	1.015, 1.106	0.0088	1.096	1.049, 1.146	< 0.0001
Abiraterone	0.363	0.314, 0.420	< 0.0001	0.392	0.339, 0.454	< 0.0001
ADT	1.242	1.171, 1.318	< 0.0001	1.282	1.202, 1.367	< 0.0001
AAT	1.059	0.995, 1.128	0.0722	1.020	0.952, 1.094	0.5657
Platinum chemotherapy	1.066	0.945, 1.203	0.3007	0.760	0.673, 0.858	< 0.0001
CVD medications^1^						
Furosemide–versus never (no before and no after)						
After PCa Dx (no before/yes after)	0.658	0.623, 0.695	< 0.0001	0.693	0.655, 0.732	< 0.0001
Before PCa Dx (yes before/no after)	1.067	0.923, 1.233	0.3799	0.719	0.622, 0.832	< 0.0001
Always (yes before/yes after)	1.508	1.393, 1.631	< 0.0001	1.210	1.116, 1.312	< 0.0001
Spironolactone–versus never (no before and no after)						
After PCa Dx (no before/yes after)	0.885	0.813, 0.964	0.0051	0.957	0.878, 1.043	0.3153
Before PCa Dx (yes before/no after)	0.913	0.713, 1.170	0.4716	0.708	0.552, 0.909	0.0068
Always (yes before/yes after)	1.358	1.146, 1.609	0.0004	1.233	1.037, 1.465	0.0174
Digoxin–versus never (no before and no after)						
After PCa Dx (no before/yes after)	1.013	0.910, 1.129	0.8087	1.006	0.903, 1.120	0.9146
Before PCa Dx (yes before/no after)	1.138	0.903, 1.434	0.2751	0.925	0.732, 1.169	0.5152
Always (yes before/yes after)	1.488	1.289, 1.716	< 0.0001	1.264	1.094, 1.461	0.0015

Abbreviations: AAT, anti‐androgen treatment; ADT, androgen deprivation therapy; CI, confidence interval; Dx, diagnosis; HR, hazard ratio; N‐H, non‐Hispanic; PCa, prostate cancer; PI, Pacific Islander; SHR, sub‐distribution hazard ratio.

^1^ After PCa Dx = Initiation; Before PCa Dx = Discontinuation; Always = Continuation.

Continuation of furosemide (HR 1.51, 95% CI 1.39–1.63), spironolactone (HR 1.36; 95% CI: 1.15–1.61), and digoxin (HR 1.49, 95% CI 1.29–1.72) after PCa diagnosis significantly increased risk of male breast cancer diagnosis in prostate cancer survivors. Initiation of furosemide (HR 0.66; 95% CI 0.62–0.70) and spironolactone (HR 0.89, 95% CI 0.81–0.96) after prostate cancer diagnosis decreased risk of male breast cancer, while discontinuation of furosemide and spironolactone after prostate cancer diagnosis did not (Table 2). Neither discontinuation nor initiation of Digoxin after prostate cancer diagnosis was significantly associated with increased risk of breast cancer.

**Figure 2:**
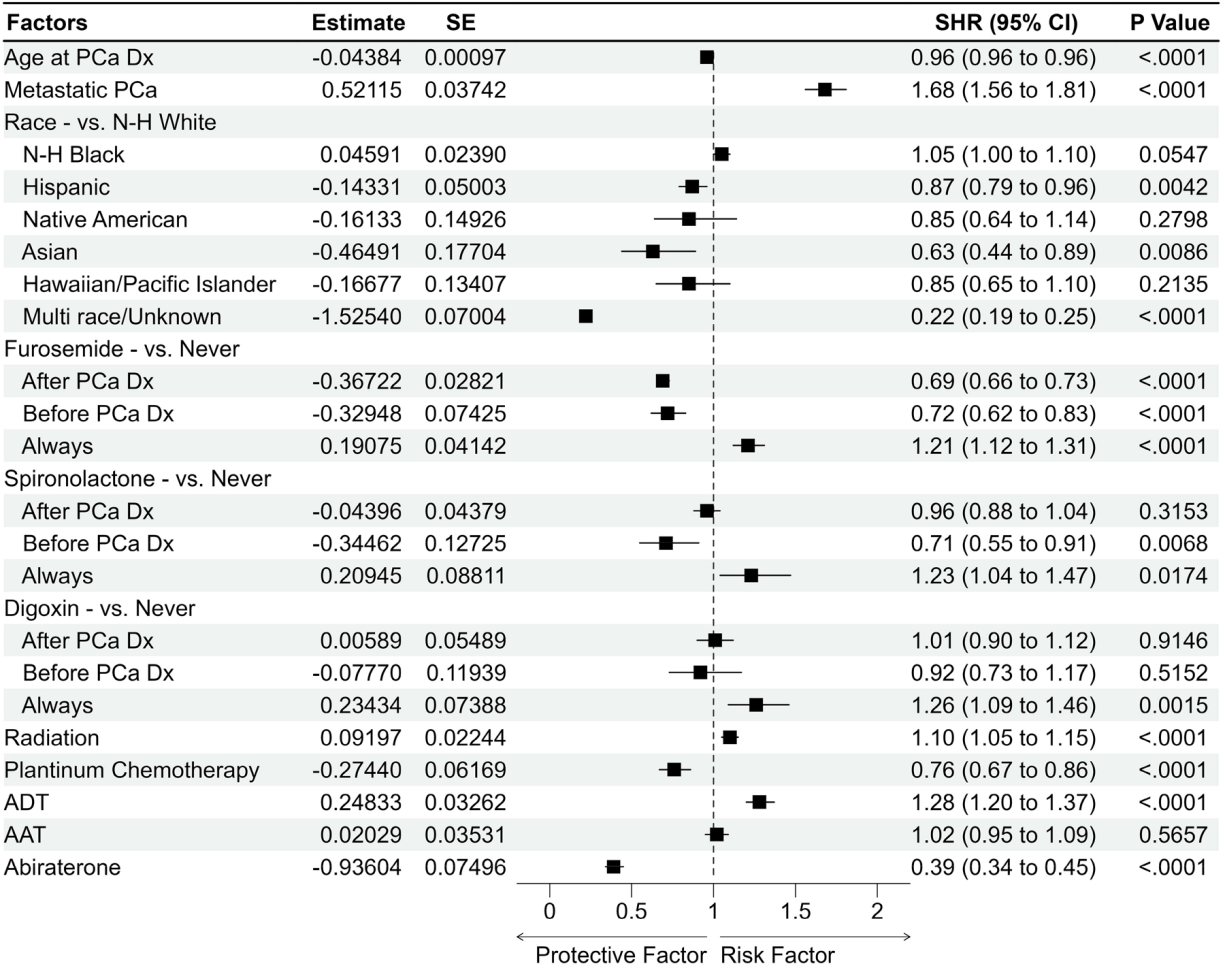
Forest plot of results from multivariable cox proportional hazards analysis on risk factors associated with male breast cancer. AAT, anti‐androgen treatment; ADT, androgen deprivation therapy; CI, confidence interval; Dx, diagnosis; N‐H, non‐Hispanic; PCa, prostate cancer; SE, standard error; SHR, sub‐distribution hazard ratio.

The competing risk model had similar results to the Cox model with younger age onset at prostate cancer, metastatic prostate cancer, brachytherapy, ADT, and digoxin as factors significantly associated with increased risk of male breast cancer in patients diagnosed with prostate cancer. Initiation of furosemide post PCa diagnosis, abiraterone, Asian, Hispanic, and multi‐race/unknown (compared to N‐H White) were significantly associated with decreased risk of male breast cancer for both Cox and competing risk models (Figure 2). N‐H Black race was a significant risk factor according to the Cox model, but its statistical significance was attenuated slightly (SHR 1.05, 95% 1.00–1.10, *p* = 0.0547) in the competing risk model. Platinum chemotherapy was not a significant risk factor according to Cox model, while it was significantly associated with decreased risk of male breast cancer according to the competing risk model (SHR 0.76, 95% CI 0.68–0.86; Table 2).

The competing risk model results, in Table 2, showed that continuation of furosemide (SHR 1.21, 95% CI 1.12–1.31), spironolactone (SHR 1.23, 95% CI 1.04–1.47), and digoxin (SHR 1.26, 95% CI 1.10–1.46) after prostate cancer diagnosis significantly increased risk of male breast cancer in prostate cancer survivors. Discontinuation or initiation of furosemide after prostate cancer diagnosis decreased risk of male breast cancer (discontinuation SHR 0.72, 95% CI 0.62–0.83; initiation SHR 0.69, 95% CI 0.66–0.73). Discontinuation of spironolactone after PCa diagnosis significantly decreased risk of breast cancer in prostate cancer survivors (SHR 0.71, 95% CI 0.55–0.91), while initiation of spironolactone after PCa diagnosis did not (Table 2). Neither discontinuation nor initiation of digoxin after prostate cancer diagnosis was significantly associated with increased risk of breast cancer. Continued use of furosemide, spironolactone, and digoxin both pre and post PCa diagnosis, were significantly associated with increased risk of male breast cancer in both Cox and competing risk models (Table 2; Figure 2).

Sensitivity analysis adding BMI as a covariate to the models was conducted and BMI was found to be not significantly associated (HR 1.00, 96% CI 1.00–1.01, *p* = 0.9288) with increased risk of male breast cancer and results of the other variables were similar to the study's final model (Supporting Information Table 4).

## 4 Discussion

Due to a rarity of patients, male breast cancer is under researched and the underlying mechanisms of it is poorly understood. While there is a lack of data, male veterans with previous prostate cancer are at an elevated risk of breast cancer (0.87%), than their civilian counterparts (0.14%) [22]. To address the current gap in knowledge and data, this study leveraged an existing large cohort of male veterans with prostate cancer and examined factors associated with increased risk of male breast cancer. Our study found younger age at diagnosis of prostate cancer, being N‐H Black male, receiving radiation (i.e., brachytherapy) and ADT during cancer treatment, continuation of furosemide, spironolactone, and digoxin after prostate cancer diagnosis, are factors that are significantly associated with increased risk of breast cancer development among male veteran prostate cancer survivors.

A younger age of onset of prostate cancer being a risk factor indicates that the likelihood of a male breast cancer diagnosis increases with the aging of prostate cancer survivors. Additionally, we found N‐H Blacks had a higher risk of breast cancer than N‐H Whites, which coincides with reports from civilian male population showing N‐H Black men with a 52% higher rate of breast cancer than N‐H White men [23].

While our finding of ADT as a risk factor for male breast cancer aligns with prior cases reports [9, 10, 24], the current study failed to find anti‐androgen treatment as a significant risk factor, which was not consistent with previous case reports [11, 12]. This is puzzling because most prostate cancer patients are treated with both anti‐androgen and ADT. Future research should examine single and dual concomitant prostate cancer treatments as risk factors for male breast cancer in prostate cancer patients.

Our study reported mixed findings about effects of chemotherapy in treating prostate cancer on increased risk of male breast cancer. While it was not significantly related to increased risk of male breast cancer in the Cox model, it was a significant protective factor in the competing risk model. This may be due to a relatively small number of prostate cancer patients treated with chemotherapy, resulting in a lack of variance.

The current study also found that continuing use of CVD medications, such as furosemide, spironolactone, and digoxin, after PCa diagnosis increased risk of breast cancer among prostate cancer survivors. This may be partly explained by patients who are continually treated with furosemide, spironolactone, and digoxin after prostate cancer diagnosis having other metabolic risk factors and are therefore more likely to have greater adverse health outcomes. While digoxin has been linked with increased risk of female breast cancer [25, 26], very few studies have examined digoxin as a risk factor for male breast cancer [18].

The current study also reported mixed findings regarding initiation of diuretics–furosemide and spironolactone—with spironolactone initiation not associated with male breast cancer risk and starting furosemide while treating prostate cancer may lower risk of male breast cancer. Prior studies have reported the use of diuretics to be associated with an increased risk of breast cancer [19] however, others have reported a lack of association [27, 28]. While the literature on the safety of concomitant use of spironolactone with prostate cancer treatments is still inconclusive [29], discontinuation of spironolactone to treat complications of heart failure or cirrhosis during cancer treatment may lower risk of later secondary cancers, such as male breast cancer.

The present study findings add to the current literature in the topic of male breast cancer, which is significantly limited due to it being a rare disease, however, there are limitations to note. First, while the current study findings are based on large‐, representative data, from male veterans with previously diagnosed prostate cancer, the results might not be generalizable to the overall male breast cancer population. Secondly, this was a retrospective cohort study, thus results may be biased, and causality is hard to establish. Thirdly, the study cohort is comprised of veterans, thus future studies in nonveteran populations are warranted. Lastly, the current study did not examine other known risk factors for male breast cancer incidence, such as family history, BRCA 2 mutations, and military environmental exposure due to lack of data. Future studies are highly warranted to examine the role of genetic predisposition on developing breast cancer among males who survived prostate cancer. Regardless of these limitations, our study is the first study that capitalized on the largest EHR database in the United States to use real world data of over one million patients, employed a competing risk model factoring in death as a competing event with new diagnosis of breast cancer in prostate cancer survivors, and identified risk factors for male breast cancer.

## 5 Conclusion

This study used real‐world EHR data from male veteran prostate cancer patients to examine risk factors for developing male breast cancer incidence and found that a younger age onset of prostate cancer, treatment with brachytherapy and ADT, and prolonged use of furosemide, spironolactone, and digoxin to treat CVD comorbid conditions, were risk factors for male breast cancer incidence. The study findings shed insights in optimal prostate cancer survivorship care by identifying risk factors for later incidence of male breast cancer which will lead to early detection and prevention.

## Ethics Statement

The study was conducted in accordance with the Declaration of Helsinki. The VA North Texas Health Care System Institutional Review Board approved the study and waived informed consent.

## Conflicts of Interest

The authors declare no conflicts of interest.

## Supporting information


**Supplemental Table 1**: ICD 9 and 10 diagnosis and CPT procedure codes for breast cancer diagnosis. **Supplemental Table 2**: ICD 9 and 10 diagnosis, CPT procedure, and ICD 9 and 10 procedure codes for prostate cancer treatments (procedures). **Supplemental Table 3:** Cardiovascular medication groups by usage (before and after prostate cancer diagnosis) and breast cancer diagnosis (*n* = 1,314,492). **Supplemental Table 4:** Supplemental analysis with BMI as a covariate (*N* = 1,241,774).

## Data Availability

Because of the sensitive nature of the data collected for this study, requests to access the individual level data are limited to qualified VA‐affiliated researchers trained in human subject confidentiality. The deidentified summary data will be available upon request. Protocols may be sent to the VA North Texas Health Care System Institutional Review Board (IRB) at NTXIRBAdmin@va.gov. SQL, SAS, and R programming code used in the analysis of this study are available from the corresponding author upon reasonable request. All methods were performed in accordance with the relevant guidelines and regulations.
