# Supplementary material for: Factors Associated With Male Breast Cancer Incidence Among Prostate Cancer Survivors: Real World Evidence From Veterans Affairs National Prostate Cancer Data Core
Source: Prostate. 2025 Oct 8;86(2):227–35. doi: 10.1002/pros.70074 (PMC12704238; doi:10.1002/pros.70074)
Supplement: Supplementary file 1 — Supplemental Table 1: ICD 9 and 10 diagnosis and CPT procedure codes for breast cancer diagnosis. Supplemental Table 2: ICD 9 and 10 diagnosis, CPT procedure, and ICD 9 and 10 procedure codes for prostate cancer treatments (procedures). Supplemental Table 3: Cardiovascular medication groups by usage (before and after prostate cancer diagnosis) and breast cancer diagnosis (n = 1,314,492). Supplemental Table 4: Supplemental analysis with BMI as a covariate (N = 1,241,774). [file PROS-86-227-s001.docx]

**Supplemental Table 1**. ICD 9 and 10 diagnosis and CPT procedure codes for breast cancer diagnosis

| **Source** | **Codes** |
| --- | --- |
| ICD 9  Diagnosis Codes | '174.0', '174.1', '174.2', '174.3', '174.4', '174.5', '174.6', '174.8', '174.9', '175.0', '175.9', '198.81', '217.', '233.0', '238.3', '239.3', '611.72', '793.8', '793.89', 'V10.3', 'V51.0', 'V52.4', 'V84.01' |
| ICD 10 Diagnosis Codes | 'C43.52', 'C44.511', 'C44.521', 'C4A.52', 'C50.011', 'C50.012', 'C50.019', 'C50.021', 'C50.022', 'C50.029', 'C50.111', 'C50.112','C50.119', 'C50.121', 'C50.122', 'C50.129', 'C50.211', 'C50.212', 'C50.219', 'C50.221', 'C50.222', 'C50.229', 'C50.311', 'C50.312','C50.319', 'C50.321', 'C50.322', 'C50.329', 'C50.411', 'C50.412', 'C50.419', 'C50.421', 'C50.422', 'C50.429', 'C50.511', 'C50.512', 'C50.519', 'C50.521', 'C50.522', 'C50.529', 'C50.611', 'C50.612', 'C50.619', 'C50.621', 'C50.622', 'C50.629', 'C50.811', 'C50.812','C50.819', 'C50.821', 'C50.822', 'C50.829', 'C50.911', 'C50.912', 'C50.919', 'C50.921', 'C50.922', 'C50.929', 'C79.81', 'D05.00','D05.01', 'D05.02', 'D05.10', 'D05.11', 'D05.12', 'D05.80', 'D24.1', 'D24.2', 'D24.9', 'D48.60', 'D48.61', 'D48.62', 'D49.3', 'S28.211A','S28.211D', 'S28.211S', 'S28.212A', 'S28.212D', 'S28.212S', 'S28.219A', 'S28.219D', 'S28.219S', 'Z15.01', 'Z42.1', 'Z44.30', 'Z44.31', 'Z44.32', 'Z85.3', 'Z86.000', 'Z90.10', 'Z90.11', 'Z90.12', 'Z90.13' |
| ICD 9  Procedure Codes | '85.11', '85.12', '85.20', '85.91', '85.95' |
| ICD 10 Procedure Codes | '0H5T0ZZ', '0H5T3ZZ', '0H5T7ZZ', '0H5T8ZZ', '0H5TXZZ', '0H5U0ZZ', '0H5U3ZZ', '0H5U7ZZ', '0H5U8ZZ', '0H5UXZZ', '0H5V0ZZ', '0H5V3ZZ', '0H5V7ZZ', '0H5V8ZZ', '0H5VXZZ', '0HHT01Z', '0HHT31Z', '0HHT71Z', '0HHT81Z', '0HHTX1Z', '0HHU01Z', '0HHU31Z', '0HHU71Z', '0HHU81Z', '0HHUX1Z', '0HHV01Z', '0HHV31Z', '0HHV71Z', '0HHV81Z', '0HHVX1Z', 'DM000ZZ', 'DM001ZZ', 'DM002ZZ', 'DM003Z0', 'DM003ZZ', 'DM004ZZ', 'DM005ZZ', 'DM006ZZ', 'DM010ZZ', 'DM011ZZ', 'DM012ZZ', 'DM013Z0', 'DM013ZZ', 'DM014ZZ', 'DM015ZZ', 'DM016ZZ', 'DM1097Z', 'DM1098Z', 'DM1099Z', 'DM109BZ', 'DM109CZ', 'DM109YZ', 'DM10B7Z', 'DM10B8Z', 'DM10B9Z', 'DM10BBZ', 'DM10BCZ', 'DM10BYZ', 'DM1197Z', 'DM1198Z', 'DM1199Z', 'DM119BZ', 'DM119CZ', 'DM119YZ', 'DM11B7Z', 'DM11B8Z', 'DM11B9Z', 'DM11BBZ', 'DM11BCZ', 'DM11BYZ', 'DM20DZZ','DM20HZZ', 'DM20JZZ', 'DM21DZZ', 'DM21HZZ', 'DM21JZZ', 'DMY0KZZ', 'DMY1KZZ' |

**Abbreviations.** ICD **=** International Classification of Diseases

**Supplemental Table 2**. ICD 9 and10 diagnosis, CPT procedure, and ICD 9 and 10 procedure codes for prostate cancer treatments (procedures)

| **Source** | **Codes** |
| --- | --- |
| ***Radiation - brachytherapy*** | |
| ICD 9  Diagnosis Codes | 'V58.0', 'V66.1', 'V67.1', 'E873.2', 'E879.2 |
| ICD 10 Diagnosis Codes | 'Z51.0', 'Z51.89', 'Z08.', 'Y63.2', 'Y84.2' |
| CPT Codes | '0182T', '19298', '21175', '57155', '57156', '58346', '76873', '77316', '77317', '77318', '77326', '77327','77328', '77781', '77782', '77783', '77784', '77785', '77786', '77787', '77799', '92974', 'C1164', 'C1712','C1799', 'C1800', 'G0256', 'G0261', 'G0458', '0073T', '0082T', '0083T', '0182T', '0190T', '0197T', '19296', '19297','19298', '20555', '20660', '31463', '32553', '333', '344', '41019', '49411', '49412', '52250', '55859', '55860', '55875', '55876', '55920','57155', '57156', '58346', '61720', '61735', '61770', '61781', '61782', '61783', '61793', '61795', '61796', '61797', '61798', '61799', '61800','63620', '63621', '73670', '76950', '76965', '77014', '77261', '77262', '77263', '77280', '77285', '77290', '77295', '77299', '77300', '77301','77305', '77306', '77307', '77310', '77315', '77321', '77326', '77326', '77327', '77327', '77328', '77328', '77331', '77332', '77333', '77334','77336', '77338', '77370', '77370', '77371', '77372', '77373', '77380', '77381', '77385', '77386', '77387', '77399', '77400', '77401', '77402','77403', '77404', '77405', '77406', '77407', '77408', '77409', '77410', '77411', '77412', '77413', '77414', '77415', '77416', '77417', '77418','77419', '77420', '77421', '77422', '77423', '77425', '77427', '77430', '77431', '77432', '77435', '77469', '77470', '77499', '77520', '77522','77523', '77525', '77750', '77761', '77762', '77763', '77776', '77777', '77778', '77781', '77782', '77783', '77784', '77785', '77786', '77787','77789', '77790', '77799', '79005', '79030', '79035', '79100', '79101', '79200', '79300', '79400', '79403', '79420', '79440', '79445', '79900','79999', 'A4650', 'A9606', 'A9699', 'C1715', 'C1716', 'C1717', 'C1718', 'C1719', 'C1720', 'C1728', 'C2616', 'C2633', 'C2634', 'C2635', 'C2636','C2637', 'C2638', 'C2639', 'C2640', 'C2641', 'C2642', 'C2643', 'C2698', 'C2699', 'C9726', 'C9728', 'G0173', 'G0174', 'G0242', 'G0243', 'G0251','G0338', 'G0339', 'G0340', 'G6003', 'G6004', 'G6005', 'G6006', 'G6007', 'G6008', 'G6009', 'G6010', 'G6011', 'G6012', 'G6013', 'G6014', 'G6015','G6016', 'Q3001', 'S2270', 'S8049', 'C1325', 'C1348', 'C1350', 'C1700', 'C1701', 'C1702', 'C1703', 'C1704', 'C1705', 'C1706', 'C1707', 'C1708','C1709', 'C1710', 'C1711', 'C1712', 'C1790', 'C1791', 'C1792', 'C1793', 'C1794', 'C1795', 'C1796', 'C1797', 'C1798', 'C1799', 'C1800', 'C1801','C1802', 'C1803', 'C1804', 'C1805', 'C1806', 'C2632', 'C9714', 'C9715', 'G0178', 'G0256', 'G0273', 'G0274', 'G0338', 'G0339', 'G0340', 'G0458','C2644', 'C2645', '55862', '55865', '4200F' , '4201F' |
| ICD 9  Procedure Codes | '92.20', '92.21', '92.22', '92.23', '92.24', '92.25', '92.26', '92.27', '92.28', '92.29', '92.3', '92.31', '92.32', '92.33', '92.39', '92.41' |
| ICD 10 Procedure Codes | 'D81099Z', 'D914B8Z', 'DB12BYZ', 'DW16BYZ', '3E0D704', '3E0J304', 'D0109YZ', 'D010BYZ', 'D0169YZ', 'D016BYZ', 'D714BYZ', 'D7189YZ', 'D81097Z', 'D9149YZ', 'D91B9YZ', 'DB11BYZ', 'DB129YZ', 'DB12BYZ', 'DB16BYZ', 'DB17BYZ', 'DD109YZ', 'DD10B7Z', 'DD14BYZ', 'DD17BYZ', 'DF109YZ', 'DF10BYZ', 'DG149YZ', 'DG1599Z', 'DG15B9Z', 'DG15BYZ', 'DT1299Z', 'DT129YZ', 'DT12BYZ', 'DU11BYZ', 'DV1099Z', 'DV10B9Z', 'DV10BBZ', 'DV10BYZ', 'DW119YZ', 'DW129YZ', 'DW12BYZ', 'DW13BYZ', '0VH001Z' , '0VH031Z' , '0VH041Z' , '0VH071Z' , '0VH081Z' , '0VP481Z' , 'DV000ZZ' , 'DV001ZZ' , 'DV002ZZ' , 'DV003Z0' , 'DV003ZZ' , 'DV004ZZ' , 'DV005ZZ' , 'DV20DZZ' , 'DV20JZZ' , 'DVY07ZZ' , 'DVY0CZZ' , 'DVY0FZZ' |
| ***ADT^1^ - orchiectomy*** | |
| CPT Codes | '54520', '54522', '54530', '54535' |
| ICD 9  Procedure Codes | '62.3'OR '62.4%' |
| ICD 10 Procedure Codes | '0VT90ZZ', '0VT94ZZ', '0VTB0ZZ', '0VTB4ZZ', '0VTC0ZZ', '0VTC4ZZ' |

**Abbreviations.** ICD **=** International Classification of Diseases; CPT = Current Procedural Terminology; ADT = Androgen Depravation Therapy

**Notes**. 1. ADT includes: orchiectomy (procedure) and/or leuprolide, goserelin, triptorelin, histrelin, buserelin, degarelix, and relugolix (medications). Medications were identified using drug names in inpatient, outpatient, and fee-based pharmacy data.

**Supplemental Table 3.** Cardiovascular medication groups by usage (before and after prostate cancer diagnosis) and breast cancer diagnosis (n=1,314,492)

| **Pre / Post PCa Dx^1^** | | **Furosemide** | | | **Spironolactone** | | | **Digoxin** | | |
| --- | --- | --- | --- | --- | --- | --- | --- | --- | --- | --- |
|  |  | No BCa  n=1,303,165 | BCa  n=11,327 | All (100%) | No BCa  n=1,303,165 | BCa  n=11,327 | All (100%) | No BCa  n=1,303,165 | BCa  n=11,327 | All (100%) |
| **No / No** | n (%) | 975,620  (74.87) | 8,630 (76.19) | 984,250 (74.88) | 1,215,920  (93.31) | 10,499  (92.69) | 1,226,419 (93.30) | 1,230,206  (94.40) | 10,689  (94.37) | 1,240,895 (94.40) |
|  | Duration in months  (M±SD) | 0 ± 0^†^ | | | 0 ± 0^‡^ | | | 0 ± 0^§^ | | |
| **No / Yes** | n (%) | 217,859  (16.72) | 1,679  (14.82) | 219,538 (16.70) | 63,575  (4.88) | 615  (5.43) | 64,190 (4.88) | 43,199  (3.31) | 356  (3.14) | 43,555 (3.31) |
|  | Duration in months  (M±SD) | 33.44±  43.14 | 71.29±  64.01 | 33.73 ± 43.47† | 23.75±  32.86 | 44.67±  49.00 | 23.96 ± 33.12‡ | 30.82±  38.21 | 55.96±  53.77 | 31.03 ± 38.43§ |
| **Yes / No** | n (%) | 35,967  (2.76) | 192  (1.70) | 36,159 (2.75) | 11,713  (0.90) | 65  (0.57) | 11,778 (0.90) | 10,789  (0.83) | 74  (0.65) | 10,863 (0.83) |
|  | Duration in months (M±SD) | 27.17±  41.96 | 18.53±  27.80 | 27.12 ± 41.90† | 21.96±  32.65 | 17.65±  25.75 | 21.93 ± 32.61‡ | 26.52±  36.54 | 10.37±  14.46 | 26.41 ± 36.46§ |
| **Yes / Yes** | n (%) | 73,719  (5.66) | 826  (7.29) | 74,545 (5.67) | 11,957 (0.92) | 148 (1.31) | 12,105 (0.92) | 18,971  (1.46) | 208  (1.84) | 19,179 (1.46) |
|  | Duration in months (M±SD) | 96.75±  66.90 | 128.13±  71.16 | 97.10 ± 67.02† | 82.21±  61.54 | 87.60±  64.59 | 82. 27 ± 61.58‡ | 79.22±  57.03 | 90.99±  58.52 | 79.35 ± 57.06§ |

**Abbreviations.** PCa Dx= prostate cancer diagnosis; BCa = breast cancer; M = mean; SD = standard deviation

**Notes.**

1. Pre/Post PCa Dx: No / No = Never (Not taking before or after PCa Dx); No / Yes = Initiation –After PCa only (New medication started on PCa Dx or after); Yes / No = Discontinuation – Before prostate cancer only (taking before, but discontinued after or on PCa Dx); Yes / Yes = Continuation – Always (taking before and after PCa Dx)

† Welch Anova (Levene’s test significant- unequal variances) significant, p-value < 0.0001, and duration between all four furosemide medication usage groups different at 0.05 alpha level.

‡ Welch Anova (Levene’s test significant- unequal variances) significant, p-value < 0.0001, and duration between all four spironolactone medication usage groups different at 0.05 alpha level.

§ Welch Anova (Levene’s test significant- unequal variances) significant, p-value < 0.0001, and duration between all four digoxin medication usage groups different at 0.05 alpha level.

**Supplemental Table 4.** Supplemental analysis with BMI as a covariate (N=1,241,774)

| **Variables** | **Cox with BMI** | | | **Competing Risk with BMI** | | | | |  |
| --- | --- | --- | --- | --- | --- | --- | --- | --- | --- |
|  | **HR** | **95% CI** | **P-value** | **SHR** | **95% CI** | | **P-value** | |  |
| *Covariates* |  |  |  |  |  | |  | |  |
| Age PCa Dx | 0.974 | 0.972, 0.976 | <.0001 | 0.958 | 0.956, 0.960 | | <.0001 | |  |
| Metastatic PCa Dx | 2.035 | 1.905, 2.173 | <.0001 | 1.698 | 1.578, 1.828 | | <.0001 | |  |
| BMI^1^ | 1.000 | 0.996, 1.003 | 0.9288 | 1.006 | 1.003, 1.010 | | 0.0005 | |  |
| Race – vs. N-H White | | | |  |  | |  | |  |
| Native American | 0.874 | 0.653, 1.170 | 0.3663 | 0.852 | 0.636, 1.141 | | 0.2820 | |  |
| Asian | 0.610 | 0.432, 0.861 | 0.0050 | 0.637 | 0.450, 0.901 | | 0.0108 | |  |
| Hawaiian/PI | 0.817 | 0.629, 1.061 | 0.1300 | 0.841 | 0.647, 1.094 | | 0.1968 | |  |
| Hispanic | 0.842 | 0.763, 0.928 | 0.0006 | 0.863 | 0.782, 0.952 | | 0.0033 | |  |
| Multi race/  Unknown | 0.402 | 0.348, 0.465 | <.0001 | 0.329 | 0.285, 0.380 | | <.0001 | |  |
| N-H Black | 1.096 | 1.046, 1.148 | 0.0001 | 1.056 | 1.007, 1.106 | | 0.0241 | |  |
| *PCa Treatments* |  |  |  |  |  | |  | |  |
| Radiation Therapy | 1.043 | 0.999, 1.089 | 0.0543 | 1.076 | 1.030, 1.124 | | 0.0011 | |  |
| Abiraterone | 0.358 | 0.310, 0.415 | <.0001 | 0.385 | 0.332, 0.446 | | <.0001 | |  |
| ADT | 1.244 | 1.172, 1.320 | <.0001 | 1.278 | 1.199, 1.362 | | <.0001 | |  |
| AAT | 1.055 | 0.991, 1.123 | 0.0955 | 1.014 | 0.946, 1.087 | | 0.6989 | |  |
| Platinum Chemotherapy | 1.064 | 0.943, 1.201 | 0.3125 | 0.764 | 0.677, 0.863 | | <.0001 | |  |
| *CVD Medications^1^* |  |  |  |  |  | |  | |  |
| Furosemide – vs. Never (No Before & No After) | | | |  | |  | |  | |
| After PCa Dx  (No Before/Yes After) | 0.656 | 0.620, 0.693 | <.0001 | 0.679 | 0.643, 0.718 | | <.0001 | |  |
| Before PCa Dx  (Yes Before/No After) | 1.062 | 0.919 1.228 | 0.4143 | 0.703 | 0.608, 0.814 | | <.0001 | |  |
| Always  (Yes Before/Yes After) | 1.503 | 1.387, 1.628 | <.0001 | 1.170 | 1.078, 1.270 | | 0.0002 | |  |
| Spironolactone – vs. Never (No Before & No After) | | | |  |  | |  | |  |
| After PCa Dx  (No Before/Yes After) | 0.882 | 0.810, 0.960 | 0.0038 | 0.945 | | 0.867, 1.029 | | 0.1942 | |
| Before PCa Dx  (Yes Before/No After) | 0.911 | 0.711, 1.167 | 0.4624 | 0.702 | | 0.547, 0.901 | | 0.0055 | |
| Always  (Yes Before/Yes After) | 1.356 | 1.144, 1.607 | 0.0004 | 1.222 | | 1.029, 1.453 | | 0.0225 | |
| Digoxin – vs. Never (No Before & No After) | | | |  |  | |  | |  |
| After PCa Dx  (No Before/Yes After) | 1.000 | 0.897, 1.115 | 0.9935 | 0.997 | | 0.895, 1.111 | | 0.9581 | |
| Before PCa Dx  (Yes Before/No After) | 1.134 | 0.900, 1.429 | 0.2863 | 0.926 | | 0.733, 1.170 | | 0.5176 | |
| Always  (Yes Before/Yes After) | 1.482 | 1.284, 1.710 | <.0001 | 1.274 | | 1.102, 1.472 | | 0.0011 | |

**Abbreviations.** HR = Hazard Ratio; CI= Confidence Interval; PCa = Prostate Cancer; Dx = Diagnosis; BMI = Body Mass Index; N-H = Non-Hispanic; PI = Pacific Islander; ADT = Androgen Depravation Therapy; AAT = Anti-Androgen Treatment

1. BMI missing for total sample is 71,718 (5.46%). Complete case analysis was conducted and patients with missing BMI were excluded from Cox and Competing Risk models.
